# Quality of Postoperative Recovery in Patients Undergoing Video Laparoscopy Cholecystectomy in a University Hospital

**DOI:** 10.7759/cureus.80234

**Published:** 2025-03-07

**Authors:** Raphael K Confessor de Sousa, Hugo W Araújo, Juliana M Freire, Matheus H De Almeida Ribeiro, Ricardo F Arrais

**Affiliations:** 1 Anesthesiology, Onofre Lopes University Hospital, Natal, BRA; 2 Anesthesiology and Perioperative Medicine, Onofre Lopes University Hospital, Natal, BRA; 3 Anesthesiology, Universidade Federal do Estado do Rio Grande do Norte, Natal, BRA; 4 General Practice, Universidade Federal do Estado do Rio Grande do Norte, Natal, BRA; 5 Pediatric Endocrinology, Universidade Federal do Estado do Rio Grande do Norte, Natal, BRA

**Keywords:** laparoscopic cholecystectomy, patient-reported outcome measures, qor-15, quality of care, quality of recovery

## Abstract

Postoperative recovery (PR) is a complex, multifactorial process, resulting largely from the confluence of physical, physiological, and psychological factors. The Quality of Recovery-15 (QoR-15) questionnaire is a tool used to assess patient's satisfaction with their recovery after surgery. The primary objective of the study is to use the QoR-15 questionnaire to describe and compare the preoperative and 24-hour postoperative scores in patients undergoing elective laparoscopic cholecystectomy (ELC) surgery. This is a cross-sectional, single-center study carried out on patients undergoing ELC, aged 18-70 years, and classified as ASA Physical Status I-III. Patients were invited to answer the QoR-15 during hospitalization, preoperatively, and the next day after the procedure. Data collected included age, sex, weight, height, preoperative fasting time, anesthesia technique, analgesic use (intraoperative and postoperative), duration of anesthesia and surgery, and antiemetic use. Data normality was checked using the Kolmogorov-Smirnov test. Wilcoxon, Fisher's exact, Kruskal-Wallis, and Mann-Whitney tests were used when appropriate. Data from 116 patients were analyzed. In our hospital, the postoperative QOR-15 was 136 (IQR: 142.0-121.7), scoring in the "excellent" category. However, there was no significant difference in relation to the preoperative QoR-15 score of 135 (IQR: 143.0-125.0), p = 0.4803. When the postoperative scores were analyzed categorically, statistically significant differences were observed for the variables of gender (p = 0.0052) and anesthetic technique (p = 0.0462). This study demonstrates that patients undergoing ELC achieved excellent postoperative recovery. Gender and anesthetic techniques appear to influence recovery quality. Further multicenter studies are required to validate these findings in diverse populations and surgical contexts.

## Introduction

Postoperative recovery (PR) is a process involving physical, psychological, and physiological factors to reestablish the patient's preoperative status [1]. Traditionally, PR has been evaluated based on specific biological, physiological, or other clinical outcomes, such as mortality, length of hospital stay, and recovery of pulmonary or renal function. Despite their importance, these variables do not always reflect patient satisfaction [2].

In line with this approach, anesthesia practitioners typically emphasize indicators related to outcomes concerning the immediate postoperative period, such as the restoration of physiological functions (e.g., arousal, respiration, thermoregulation, and management of nausea and vomiting) and the utilization of hospital resources, including length of stay and admission to intensive care units [3].

To assess PR prior to discharge from the post-anesthesia care unit (PACU), the modified Aldrete-Kroulik score can be used [4]. For the intermediate phase, which corresponds to the period from admission to the ward until hospital discharge, one of the tools used is the Quality of Recovery (QoR) Scale. In the late phase, after hospital discharge, assessments include quality of life scores and measurement of daily life activities, such as the six-minute walk test, the Community Health Activities Model Program for Seniors, the Short Form Six Dimensions (SF-6D) or the EuroQol Five Dimensions (EQ-5D) [2,5,6].

Evaluating the effectiveness of interventions designed to enhance patient satisfaction after procedures requires a patient-centered approach. The health team is responsible for optimizing recovery and can utilize tools that assess aspects of physical and mental comfort [7]. The QoR-15 is a PR assessment instrument that produces results ranging from 0-150 (0 being a poor recovery and 150 being an excellent recovery), with a response time of approximately three minutes. It presents five dimensions to be assessed in PR: pain, physical comfort, physical independence, psychological support, and emotional state [8]. The original version created in English has been translated into several languages, including Portuguese, and validated as a reliable and responsive patient-centred outcome metric [9,10].

The primary objective of this study is to use the QoR-15 tool to measure and compare preoperative and 24-hour postoperative scores in patients undergoing elective laparoscopic cholecystectomy (ELC) at a university hospital. As a secondary evaluation, we seek to describe and analyze the relationship between the QoR-15 score and anesthetic, surgical, and patient factors.

## Materials and methods

A cross-sectional study was conducted with 132 patients undergoing ELC at the Onofre Lopes University Hospital (HUOL), located in Natal, Brazil, from September 2022 to July 2024. The project was approved by the institution's ethics committee, under validation number 5.621.709 and ethical clearance certificate 61280522.0.0000.5292 (September 2nd, 2022).

The sample size was estimated to detect differences between preoperative and postoperative QoR-15 scores of 14 points, considering a standard deviation of 40 points, a statistical power of 95%, and an alpha error of 5% [11]. A total of 110 patients was required. To mitigate a potential 20% loss, data collection was planned for 132 patients.

Patients who were scheduled to undergo elective laparoscopic cholecystectomy were approached and invited to participate in the study. If accepted, they signed the informed consent form (ICF). The inclusion criteria were patients aged between 18 and 70 years with ASA physical status I-III [12]. The study excluded patients who did not consent to participate or withdrew their consent, pregnant women, individuals with cognitive impairments or neurological conditions that prevented them from understanding the questionnaire, and those requiring postoperative monitoring in the intensive care unit (ICU).

Patients completed a printed version of the QoR-15 assessment upon admission to the surgical center in the immediate preoperative period and again the next day, within 24 hours after the procedure. Additional data were collected, including age, sex, weight, height, body mass index (BMI), preoperative fasting time, anesthesia technique (general, regional, or combined), analgesic medications used intra- and postoperatively, duration of anesthesia and surgery, type of procedure and use of antiemetics. Data were collected by the authors of the study or by resident physicians previously trained in the application of the questionnaire.

The variables were represented by descriptive measures, such as frequency and percentage, median, interquartile range (IQR: Q3-Q1), mean, and standard deviation. The QoR-15 information was analyzed using the nonparametric Wilcoxon test. In addition to the numerical value obtained in the total QoR-15 scores, the results were categorized as proposed by Kleif and Gögenur [13]: under 90, 90-121, 122-135, and 136-150, representing, respectively, a poor, moderate, good or excellent recovery.

Some variables were compared according to the postoperative QoR-15 score. For this analysis, Fisher's exact test was applied to categorical variables. For numerical variables, the Kolmogorov-Smirnov test was used to verify the normality of the data. When the hypothesis of normality was rejected, the Kruskal-Wallis test was used. The Mann-Whitney test was used to verify whether the variables age, weight, and BMI presented higher values ​​in female patients compared to male patients.

For all analyses, a significance level of α = 5% was adopted. Data were processed and analyzed using R programming language (R Core Team, 2023, R Foundation for Statistical Computing, Vienna, Austria) and presented following the STROBE (Strengthening the Reporting of Observational Studies in Epidemiology) guidelines [14].

## Results

**Figure 1:**
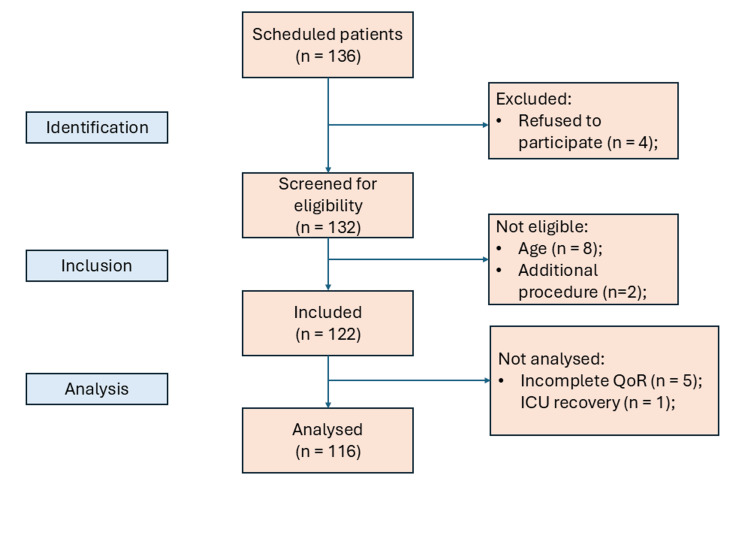
Organization Chart of Research Participants

A total of 136 patients had ECL scheduled during the research period and were invited to participate, however, only 116 were analyzed, as shown in Figure 1. Information on patient characteristics, fasting time, and duration of the procedure can be found in Tables 1-2. The patients were between 21 and 70 years old, with a mean weight of 81.3 kg (standard deviation: 20.2 kg) and a mean height of 1.6 m (standard deviation: 0.1 m). 

**Table 1:** Patient Characteristics - Part I

Variable	n (%)
Gender	
Female	98 (84.5%)
Male	18 (15.5%)
BMI classification	
Normal range	26 (22.4%)
Overweight	27 (23.3%)
Obese class I	25 (21.6%)
Obese class II	22 (19.0%)
Obese class III	16 (13.8%)
Hypertension	
Yes	47 (40.5%)
No	69 (59.5%)
Diabetes	
Yes	23 (19.8%)
No	93 (80.2%)
Thyroid disease	
Yes	7 (6.0%)
No	109 (94.0%)
ASA physical status	
ASA I	20 (17.2%)
ASA II	81 (69.8%)
ASA III	15 (12.9%)

**Table 2:** Patient Characteristics - Part II

Variable	Mean	Standard deviation
Age (years)	44.2	12.5
Weight (kg)	81.3	20.2
Height (meters)	1.6	0.1
BMI (Kg/m^2^)	31.8	7.3
Fasting time - solids (hh:mm:ss)	14:49:42	0.1514
Fasting time - liquids (hh:mm:ss)	13:07:54	0.1711
Anesthesia duration (hh:mm:ss)	03:05:03	0.0404
Surgery duration (hh:mm:ss)	02:15:19	0.0390

Among the demographic variables analyzed (age, weight, and BMI), only BMI showed a statistically significant difference between the sexes. The median BMI was 32.5 kg/m² (IQR: 37.9-26.9) in females and 27.6 kg/m² (IQR: 31.1-24.5) in males, with a p-value of 0.0246.

In the data analysis, no significant difference was observed in the total QoR-15 score between the preoperative period (135.0, IQR: 143.0-125.0) and the postoperative period (136.0, IQR 142.0-121.7) (p = 0.4803). The scores for the five dimensions assessed by the questionnaire are presented in Table 3. Significant differences were identified between the preoperative and postoperative periods in the following domains: physical independence, emotional state, and pain.

**Table 3:** QoR-15 Evaluation by Dimensions ^1^Median (IQR: Q3-Q1); ^2^Wilcoxon test

Domain	Pre-operative period^1^	Postoperative period^1^	P-value^2^
Physical comfort	46.0 (49.0-41.0)	47.0 (50.0-41.0)	0.1764
Physical independence	20.0 (20.0-18.0)	17.0 (20.0-14.0)	< 0.0001
Emotional state	33.0 (37.3-26.8)	38.0 (40.0-33.0)	< 0.0001
Psychological support	20.0 (20.0-20.0)	20.0 (20.0-20.0)	0.3849
Pain	20.0 (20.0-15.0)	16.0 (19.3-13.0)	< 0.0001

The association between the collected data and the categorized postoperative QoR-15 score can be seen in Table 4. There was statistical significance between the variables sex (p-value=0.0052) and anesthesia technique (p-value=0.0462).

**Table 4:** Variables and Postoperative QoR-15 ^1^Median (IQR: Q3-Q1);^ 2^Fisher test, Kruskal-Wallis test; NSAIDs: nonsteroidal anti-inflammatory drugs Data are presented as n (%) unless otherwise specified. Anesthesia and surgery durations are reported as median (IQR: Q3-Q1).

Variables	QoR-15				P-value^2^
	Poor	Moderate	Good	Excellent	
Sex					0.0052
Female	3 (75.0%)	25 (100.0%)	26 (92.9%)	44 (74.6%)	
Male	1 (25.0%)	0 (0.0%)	2 (7.1%)	15 (25.4%)	
Anesthesia duration - minutes^1^	175.5 (199-153)	185 (215-165)	170 (200-134)	165 (205-150)	0.2942
Surgery duration - minutes^1^	136.5 (154-119)	145 (160-125)	112.5 (144-90)	120 (160-95)	0.1506
Anesthesia techniques					0.0462
General	1 (25.0%)	14 (56.0%)	23 (82.1%)	42 (71.2%)	
General + regional anesthesia	3 (75.0%)	11 (44.0%)	5 (17.9%)	17 (28.8%)	
NSAIDs					0.5502
Parecoxib	0 (0.0%)	4 (16.0%)	9 (32.1%)	13 (22.0%)	
Tenoxicam	3 (75.0%)	18 (72.0%)	13 (46.4%)	34 (57.6%)	
No NSAIDs	1 (25.0%)	3 (12.0%)	6 (21.4%)	12 (20.3%)	
Lidocaine					0.2049
No	1 (25.0%)	9 (36.0%)	4 (14.3%)	20 (33.9%)	
Yes	3 (75.0%)	16 (64.0%)	24 (85.7%)	39 (66.1%)	
Ketamine					0.8106
No	1 (25.0%)	11 (44.0%)	10 (35.7%)	26 (44.1%)	
Yes	3 (75.0%)	14 (56.0%)	18 (64.3%)	33 (55.9%)	
Magnesium sulfate					0.7384
No	4 (100.0%)	23 (92.0%)	26 (92.9%)	50 (84.7%)	
Yes	0 (0.0%)	2 (8.0%)	2 (7.1%)	9 (15.3%)	
Clonidine					0.5852
Yes	0 (0.0%)	7 (28.0%)	8 (28.6%)	21 (35.6%)	
No	4 (100.0%)	18 (72.0%)	20 (71.4%)	38 (64.4%)	
Dexmedetomidine					1
Yes	0 (0.0%)	1 (4.0%)	1 (3.6%)	4 (6.8%)	
No	4 (100.0%)	24 (96.0%)	27 (96.4%)	55 (93.2%)	
Methadone					0.5543
Yes	1 (25.0%)	2 (8.0%)	3 (10.7%)	5 (8.5%)	
No	3 (75.0%)	23 (92.0%)	25 (89.3%)	54 (91.5%)	
Remifentanil					0.4235
No	3 (75.0%)	18 (72.0%)	18 (64.3%)	47 (79.7%)	
Yes	1 (25.0%)	7 (28.0%)	10 (35.7%)	12 (20.3%)	
Dipyrone					0.7786
No	0 (0.0%)	4 (16.0%)	6 (21.4%)	14 (23.7%)	
Yes	4 (100.0%)	21 (84.0%)	22 (78.6%)	45 (76.3%)	
Tramadol					0.4788
No	4 (100.0%)	20 (80.0%)	26 (92.9%)	53 (89.8%)	
Yes	0 (0.0%)	5 (20.0%)	2 (7.1%)	6 (10.2%)	
Intravenous morphine					0.7573
No	4 (100.0%)	23 (92.0%)	27 (96.4%)	57 (96.6%)	
Yes	0 (0.0%)	2 (8.0%)	1 (3.6%)	2 (3.4%)	
Nalbuphine					0.0974
No	3 (75.0%)	25 (100.0%)	27 (96.4%)	57 (96.6%)	
Yes	1 (25.0%)	0 (0.0%)	1 (3.6%)	2 (3.4%)	
Prophylaxis of nausea and vomiting performed intraoperatively					0.3641
One medication class	1 (25.0%)	2 (8.0%)	1 (3.6%)	6 (10.2%)	
Two or more classes	3 (75.0%)	23 (92.0%)	27 (96.4%)	53 (89.8%)	
24h postoperative analgesic medications					0.7409
As-needed prescription	0 (0.0%)	1 (4.0%)	3 (10.7%)	3 (5.1%)	
One to two time-scheduled medications.	4 (100.0%)	23 (92.0%)	25 (89.3%)	52 (88.1%)	
Three or more time-scheduled medications.	0 (0.0%)	1 (4.0%)	0 (0.0%)	4 (6.8%)	
Prophylaxis of nausea and vomiting performed 24h postoperatively					0.1724
No prophylaxis	1 (25.0%)	11 (44.0%)	15 (53.6%)	21 (35.6%)	
One medication class	2 (50.0%)	14 (56.0%)	13 (46.4%)	37 (62.7%)	
Two or more classes	1 (25.0%)	0 (0.0%)	0 (0.0%)	1 (1.7%)	

**Figure 2:**
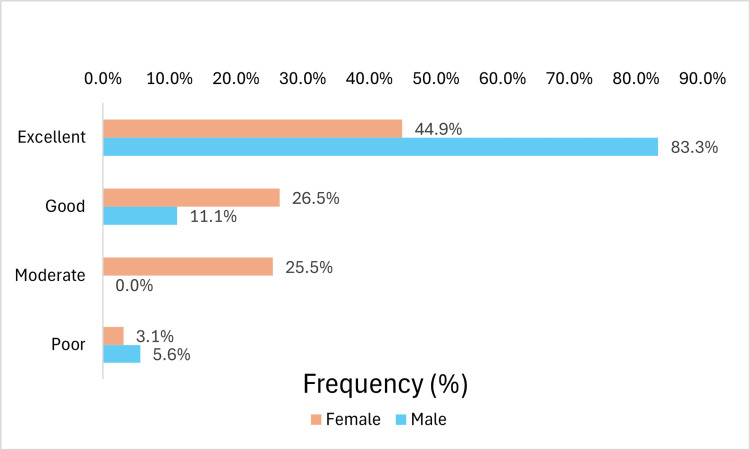
Overall Postoperative QoR-15 Scores According to Sex QoR-15: Quality of Recovery-15

Among men, 83.3% reported excellent recovery, 11.1% good, and 5.6% poor. On the other hand, among women, 44.9% had excellent recovery, 26.5% good, 25.5% moderate and 3.1% poor (Figure 2).

**Figure 3:**
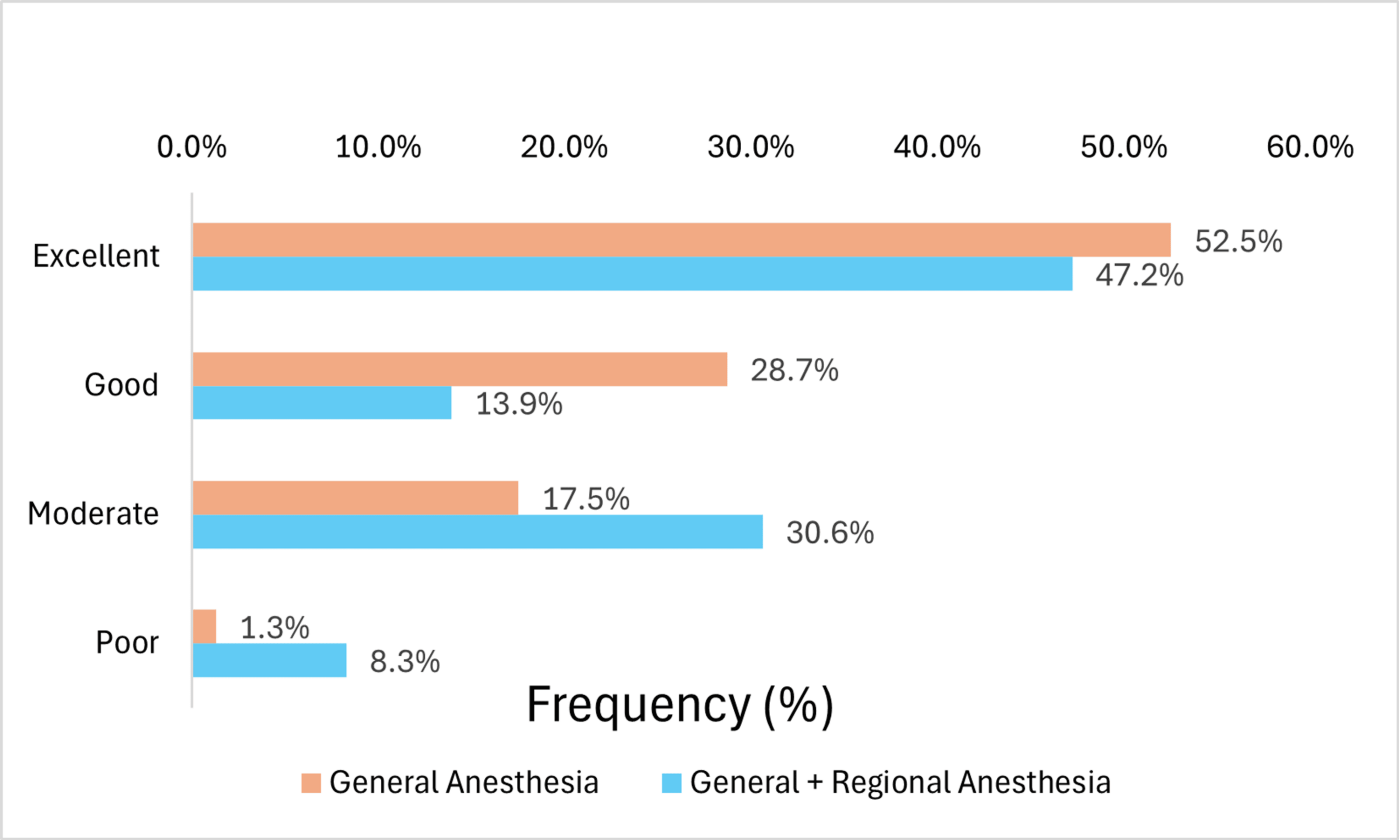
Postoperative QoR-15 Scores Categorized According to Anesthetic Techniques QoR-15: Quality of Recovery-15

Regarding the anesthesia technique, among patients who underwent general anesthesia, 52.5%, 28.7%, 17.5%, and 1.3% reported excellent, good, moderate, and poor recovery, respectively. In comparison, patients who received a combination of general and regional anesthesia reported recovery rates of 47.2%, 13.9%, 30.6%, and 8.3%, following the same order (Figure 3). 

Among the patients who received a regional block technique: 27 (75%) underwent wound infiltration, 4 (11.1%) received spinal anesthesia, 3 (8.3%) had an epidural, and 2 (5.6%) received a transversus abdominis plane block. The primary local anesthetic used was ropivacaine (80.6%), followed by bupivacaine (11.1%). The hospital's postoperative analgesic protocol included dipyrone as the first-line option and tenoxicam as the second-line option, both on a scheduled regimen. In cases of allergy to either substance, paracetamol was used as an alternative. Tramadol was the primary rescue medication for pain relief.

## Discussion

Modern PR is a multifaceted concept that extends beyond traditional metrics like mortality and hospital length of stay, emphasizing the importance of the patient’s subjective experience. Recent studies highlight that patients prioritize the restoration of daily activities, symptom management, and emotional well-being over purely clinical outcomes, such as the timing of hospital discharge [15].

Studies have demonstrated the utility of the QoR-15 across diverse surgical contexts, where it has been used in both observational studies and clinical trials to assess the impact of interventions such as peripheral nerve blocks and different anesthetic techniques [16]. The present study, conducted in a university hospital, found that the QoR-15 score in the postoperative period was 136, with no significant difference in relation to the preoperative period. However, analyzing the domains, there was an improvement in the emotional state, but a reduction in physical independence and pain, reflecting the complexity of postoperative recovery. Anxiety levels are often higher in the preoperative period, with a Brazilian study showing that over 50% of patients experience this emotion before elective procedures [17]. This may explain the lower satisfaction with emotional well-being before surgery.

It was observed that gender was a statistically significant variable in relation to postoperative QoR-15, with males presenting higher scores. Approximately 83% of male patients reported an excellent recovery, compared to 45% of females. A similar finding was reported by Kleif et al. [18] in a study of patients undergoing laparoscopic surgery for appendicitis treatment, where female patients had recovery scores approximately 21% lower than male patients on the first postoperative day. Another study concluded that sex is an independent factor in anesthesia and surgery recovery [19]. Female sex hormones, particularly progesterone, may play a role, with premenopausal women experiencing poorer overall outcomes [19]. While it is important to account for potential differences in subjective perception between sexes regarding the various domains assessed by the questionnaire, the lack of sex homogeneity represents a limitation of the present study.

Analysis of the anesthetic technique showed differences in postoperative QoR-15, with a lower score in patients who used some type of locoregional block. In the literature, the association between peripheral blocks and the quality of recovery measured by QoR-15 presents conflicting results. In breast surgeries, some studies have reported benefits in recovery with the use of peripheral blocks [20,21]. On the other hand, in minimally invasive thoracic surgeries, although a meta-analysis showed a positive effect with regional anesthesia [22], a recent randomized clinical trial found no significant advantages in using erector spinae plane block (ESP-block) for pain, opioid consumption, or QoR-15 [16]. In patients undergoing laparoscopic ventral hernia repair, the addition of an ESP block did not demonstrate an improvement in scores in a randomized, placebo-controlled clinical trial [23].

The disparity may reflect differences between surgical sites, predominance of somatic or visceral pain, and variations in the concentrations and volumes of local anesthetics. In the analysis of this study, several blockade techniques were grouped (wound infiltration, spinal anesthesia, epidural, and fascia blocks). Therefore, it was not possible to identify which of them specifically influenced the outcomes. Thus, conclusions about this association are limited.

This study has additional limitations that deserve to be noted. First, due to its observational design, it is not possible to establish causal relationships. In addition, although the questionnaire administrators were instructed to remain neutral, application bias cannot be completely excluded. The use of questionnaires also indicates the potential for recall bias. Finally, this is a single-center study, with assessment of postoperative recovery limited to the first 24 hours after the procedure, in a public university hospital in a developing country, where socioeconomic and cultural factors may influence the results. Therefore, extrapolation of the findings to other populations should be done with caution.

## Conclusions

In this analysis, most patients reported an excellent or good postoperative recovery; however, when compared to preoperative scores, there was no overall statistical difference. Among the perioperative factors evaluated, the variables sex and anesthetic technique showed an association with the quality of recovery perceived by the patients. Due to the observational nature of the study and the limitations pointed out, it is understood that further studies are necessary to elucidate and extrapolate the data presented.
